# The effects of fluoxetine on attachment and righting behaviours in marine (*Gibbula unbilicalis*) and freshwater (*Lymnea stagnalis*) gastropods

**DOI:** 10.1007/s10646-018-1919-3

**Published:** 2018-03-09

**Authors:** Alex T. Ford, Bernice Hyett, Daniel Cassidy, Graham Malyon

**Affiliations:** Institute of Marine Sciences, School of Biological Sciences, University of Portsmouth, Ferry Road, Portsmouth, PO4 9LY UK

**Keywords:** Antidepressants, Behaviour, SSRI, Mollusca, Gastropoda

## Abstract

Recent studies have highlighted that antidepressants such as the selective serotonin reuptake inhibitors (SSRIs) entering aquatic systems through wastewater discharges might impact organisms at environmentally relevant concentrations. In this study, two snail species (*Gibbula unbilicalis* and *Lymnea stagnalis*) representing the marine and freshwater environments were exposed to a large range of fluoxetine concentrations (1 ng L^−1^—1 mg L^−1^) and two distinct behaviours (foot detachment and righting time) were recorded. Fluoxetine significantly caused foot detachment only at the higher of the concentrations (1 mg L^−1^) in both species during the course of this short term 1.5 h and 4 h exposures. In this study, lowest observed effect concentrations (LOECs) for foot detachment fell repeatedly within the range for other gastropod snails exposed to fluoxetine. Fluoxetine effected righting times in a concentration dependant manner but only significantly within *G. unbilicalis* in the highest concentration. Reviewing existing data on the effects of antidepressants on a range of endpoints in gastropod molluscs reveals wide variability of results. The importance of publishing ‘negative’ and/or non-dramatic results to aid risk assessment are discussed along with the variability between antidepressants, model species, experimental designs and endpoints.

## Introduction

Antidepressants have been ranked highly amongst those pharmaceuticals with a potential risk to the aquatic environment based on their biological activity, existing toxicity data and widespread use/detection (Muñoz et al. 2008; Sanderson et al. 2004; Alonso et al. 2010; Cooper et al. 2008; Donnachie et al. 2016). Selective Serotonin Reuptake Inhibitors (SSRIs) inhibit the reuptake of serotonin from synaptic cleft thereby increasing the signals between neurones. Fong and Ford (2014) report that serotonin is an important neurohormone in invertebrates controlling many biological functions including growth, immunity, reproduction, metabolism as well as behaviour. In their review, they highlight that since the mode of action of antidepressants is by modulating the neurotransmitters serotonin, dopamine, and norepinephrine, aquatic invertebrates who possess transporters and receptors sensitive to activation by these pharmaceuticals are potentially affected by them (Fong and Ford 2014). In addition, Ford and Fong (2016) highlight that many SSRIs don’t only act upon the reuptake proteins but also actively bind to multiple neurological receptors (Stahl 1998) increasing the potential for variable downstream physiological effects.

The concentrations of antidepressants detected in the aquatic environment vary based on drug and country or even region whereby they are prescribed. One of the most commonly prescribed antidepressants (Wong et al. 1995) is fluoxetine (Prozac) for which arguably the most amount of ecotoxicological data exists. Over the past decade there has an increasing number of studies, which have reporting that antidepressants can impact the normal functioning of aquatic organisms at environmentally relevant concentrations (see Fong and Ford 2014; Ford and Fong 2016). Several studies have detected environmentally relevant concentrations of fluoxetine ranging between 0.012 and 0.54 µg L^−1^ in freshwater environments (Weston et al. 2001; Kolpin et al. 2002; Chen et al. 2006; Gardner et al. 2012). For example, Kolpin et al. (2002) measured fluoxetine at 0.012 µg L^−1^ downstream from wastewater treatment plants and Weston et al. (2001) from 0.32 to 0.54 µg L^−1^ in municipal effluent. Concentrations of fluoxetine in the estuary of Long Island Sound (New York City) have been recorded at 0.7 ± 0.3 ng L^−1^ following an approximate 80% removal rate from the wastewater treatment plant (Influent 144 ng L^−1^; Effluent 27 ng L^−1^; Lara-Martín et al. (2014)).

Within the Mollusca, antidepressants have been reported to effect behaviour, reproduction, immunity and cognitive ability (Fong et al. 1998; Fong and Molnar (2008); Fong and Hoy 2012; Di Poi et al. 2013; Bidel et al. 2016; Munari et al. 2014; Minguez et al. 2014; Peters and Granek 2016). Notably, is hugely variable data on the effects of these compounds between closely related species and drugs with ‘similar’ modes of action (see Ford and Fong 2016 for discussion on the variable receptor activity of antidepressants). However, limitations in study design and variability have led to speculation over the repeatability of the studies (Sumpter et al. 2014).

Recently there has been renewed interested in the role of behavioural toxicology partly spurred on by rapid technological advances and the ability to quantify behaviours in a high-throughput manner (Pyle and Ford 2017). One intriguing study, elegant by the relative low cost and simplicity of design is the foot detachment by gastropod molluscs. Any detachment from normal areas of shelter or feeding by a gastropod snail in their natural environment might be considered individually damaging both in terms of energy used in relocation, lost feeding times or increased predatory risks (Lemmnitz et al. 1989). Based on the knowledge that monoamine neurotransmitters are been known to impact foot attachment in snails (Sakharov and Salanki 1982), Fong and Hoy (2012) exposed two freshwater gastropod species (*Leptoxis carinata* and *Stagnicola*(*Lymnaea*) *elodes*) to the selective serotonin and norepinephrine inhibitor (SNRIs), venlafaxine and the SSRI, citralopram. They recorded the time for snails to detach from the sides of their tanks over a 4 h period. They recorded a linear concentration based response with lowest observed effect concentrations within the 31.3–313 pg L^−1^ range for *L. carinata*. The results, however, were quite different for *S. elodes* with LOECs for the SSRI (4.05 µg L^−1^) and the SNRI (31.3 ng L^−1^), thus many orders of magnitude different. As a follow-up Fong and Molnar (2013) using five different marine gastropods (*Chlorostoma funebralis*, *Nucella ostrina*, *Urosalpinx cinerea*, *Tegula fasciatus* and *Lithopoma americanum*) and four different antidepressants (fluoxetine, fluvoxamine, venlafaxine and citralopram) again observed some quite variable results. The authors highlighted the considered differences (pg–mg L^−1^) in the sensitivities of the freshwater snails in Fong and Hoy (2012) and the marine snails reported in Fong and Molnar 2013. Markedly, were the variable sensitivities between the snail families with the trochids and turbinids snails were 2–10x more sensitive to the antidepressants than the muricid snails, which the authors suggested maybe due to difference of the physiological mechanisms of locomotion (Fong and Molnar 2013).

Venlafaxine and fluoxetine have also been reported to alter crawling speed and time to reach a water interface in different ways in the two marine snails *U. cinerea* and *L. americanum* (Fong et al. 2015). Venlafaxine was reported to have sped up locomotion behaviours whilst fluoxetine slowed them down with LOECs recorded between 31.3 and 345 µg L^−1^. In their most recent study, Fong et al. (2017) recorded the ‘righting time’ (time taken to fully right following upside-down placement) in the marine snail *Llyanassa obsolete* when exposed to four different antidepressants (fluoxetine, sertraline, paroxetine and venlafaxine) with lowest concentration to show an effect being 3.45 µg L^−1^ fluoxetine.

Given the variability observed in results to date and the need to ascertain the risk posed by those kinds of contaminants, the aim of this study was to compare the effects of fluoxetine on foot detachment and righting times in further species of marine and one freshwater gastropods. This study had three main objectives: (1) increase the available data on this topic for risk assessment (2) test the repeatability within our experiments (3) compare intra/interspecies variability between a marine and freshwater snail. The chosen species were *Gibbula unbilicalis* (marine flat top shell) and *Lymnea stagnalis* (freshwater) both of which are widely found across Europe. The top shell can be found intertidally across Western Europe and the Western Mediterranean. *Lymnea stagnalis* (Great Pond Snail) is Holarctic in distribution and is widely used as a model species in neurobiology. In addition, we conducted a mini review of lowest observed effect data for foot detachment behaviours to determine variability between antidepressants.

## Methods

All *Gibbula unbilicalis* were collected outside the Institute of Marine Sciences (Langstone Harbour, Portsmouth, UK) during 2016 and 2017 and kept in external flow through tanks for a minimum of 7 days prior to experiments. External flow through tanks receive natural seawater (pH 8.1) from Langstone Harbour, which is filtered through a 4-weir sedimentation system following by glass bead and sand filtration at ambient temperatures. The seawater system is connected to heater-chillers and tanks kept were temperature controlled rooms. The fluoxetine concentrations in Langstone Harbour are not known therefore field collected specimens may have been exposed to effluent periodically from storm water overflows. All *Lymnea stagnalis* were purchased from a commercial supplier and kept within the laboratory in artificial pondwater for at least 7 days prior to experiments to acclimate to the conditions and eliminate any individuals in poor health. All fluoxetine hydrochloride (CAS number 56296-78-7) stock solutions (1 mg L^−1^, 4 mg L^−1^ or 10 mg L^−1^) were made up either in seawater or freshwater without the use of solvents and serially diluted in volumetric flasks to test solutions.

### Experiment 1

Forty* G. umbilicalis* were collected and following an acclimation period (see above) were exposed to either 1 ng, 1 µg, 1 mg L^−1^ fluoxetine or a natural filtered seawater control (pH 8.1; 21 ± 1 °C). Ten specimens per treatment were placed carefully into 500 ml beakers containing control seawater and after attachment ( < 30 min), proportions of the 10 mg L^−1^ stock were slowly decanted/pipetted into the beakers, the solutions carefully stirred and the time recorded for the snails to detach recorded. Each observation lasted 90 min. The experiment was repeated three times using separate specimens (*n* = 3 × 40 = 120).

### Experiment 2

Sixty (ten per treatment) *Gibbula umbilicalis* and *Lymnea stagnalis* were acclimated for 1 week in artificial seawater made up from reverse osmosis (RO) water (Tropic Marine® 35.2‰ pH 8.1) or RO water (pH 6.7) at 24 ± 1 °C. Experimental exposures took place in 250 ml Pyrex beakers whereby snails were gently added to beakers containing 100 ml marine or freshwater solutions (control solutions) to allow them to attach to the sides of the beaker. This would normally take <30 min and any individuals not attaching within this time were removed and replaced. Once attached an additional 100 ml of test solution was slowly decanted into the beakers making up test solution concentrations of 0 (control), 1 ng L^−1^, 10 ng L^−1^, 100 ng L^−1^, 1 µg L^−1^ and 10 µg L^−1^ fluoxetine hydrochloride concentrations. Beakers were randomly assorted and monitored over a 4 h period and the number of organisms detaching noted.

### Experiment 3

Thirty (ten per treatment) *Gibbula umbilicalis* and *Lymnea stagnalis* were acclimated for 1 week in artificial seawater (Tropic Marine® 35.2‰ pH 8.1) or RO water (pH 6.7) at 24 ± 1 °C. Experiments followed the same procedures as experiment number 2 apart from concentrations of fluoxetine were increased to 0.01 mg L^−1^ and 1 mg L^−1^ and the righting time was recorded prior to and after the 4 h exposure period. The snails were completely inverted with its orifice pointing upwards and the snails were considered completely righted when its foot was firmly attached to the substrate (as per Fong et al. 2017).

### Comparison of foot detachment data

Lowest observed effects concentrations (LOECs) for foot detachment with four antidepressants (venlafaxine, citralopram, fluoxetine and fluvoxamine) representing nine species of snails were compiled from existing published data and results from this paper.

### Data analysis

Where repeated studies were conducted (Exp 1) means were compared by a non-parametric Kruskal–Wallis test followed by Bonferroni corrected Mann–Whitney *U*-tests. Foot detachment experiments in experiment 3 were analysed using binary logistic regression and mean time to detach were analysed by a Mann–Whitney *U*-test. The relationship between snail size and pre and post righting times were analysed by Pearson’s correlation and the mean time for snails to ‘right’ themselves was analysed by Kruskal–Wallis test followed by Bonferroni corrected Mann–Whitney *U*-tests.

## Results

### Experiment 1

**Fig. 1:**
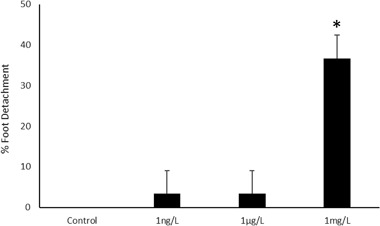
Percent ( ± 2 SE) *G. umbilicalis* displaying foot detachment exposed to 0, 1 ng L^−1^, 1 µg L^−1^ and 1 mg L^−1^ fluoxetine hydrochloride over a 1.5 h exposure period (*represents significantly different from the control *p* < 0.05)

No *G. umbilicalis* from control treatments detached during the 90 min recording period and only 1 from 30 individuals in both the 1 ng L^−1^ and 1 µg L^−1^ Flx concentrations. Significant differences were observed in the mean percentage of snails, which detached from the three repeated experiments (Kruskal–Wallis; *χ* = 8.25, df = 3, *p* = 0.041; Fig. 1). Multiple comparison tests (with Bonferroni adjustment) revealed the only significant difference occured between the control and the highest concentration (*p* = 0.046).

### Experiment 2

No snails (0/120) detached for either species during the extended monitoring period of 4 h for the lower concentrations 1 ng–10 µg L^−1^ (data not shown).

### Experiment 3

**Fig. 2:**
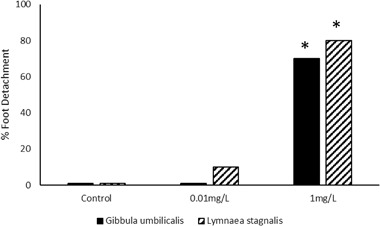
Percent *G. umbilicalis* and *L. stagnalis* displaying foot detachment exposed to 0, 0.01 mg L^−1^, and 1 mg L^−1^ fluoxetine hydrochloride over a 4 h exposure period (* represents significantly different from the control *p* < 0.05)

**Fig. 3:**
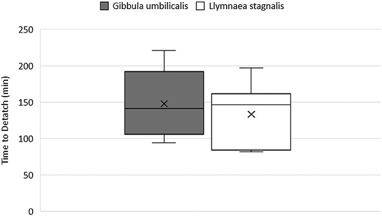
Mean time (min) to detach from tanks in *G. umbilicalis* and *L. stagnalis* exposed to 1 mg L^−1^ fluoxetine hydrochloride. (*x* = mean; line = median; bars = min/max; boxes = 25th/75th percentile)

**Fig. 4:**
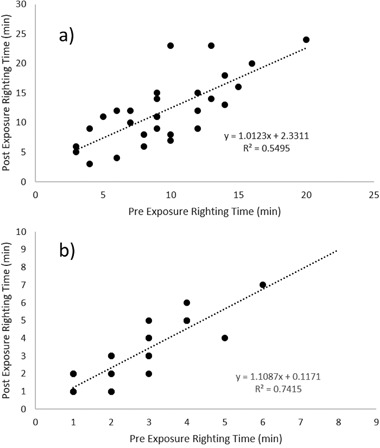
Correlation between pre and post-exposure righting times (mins) in **a** the marine snail *G. umbilicalis* and **b** in the freshwater snail *L. stagnalis*

**Fig. 5:**
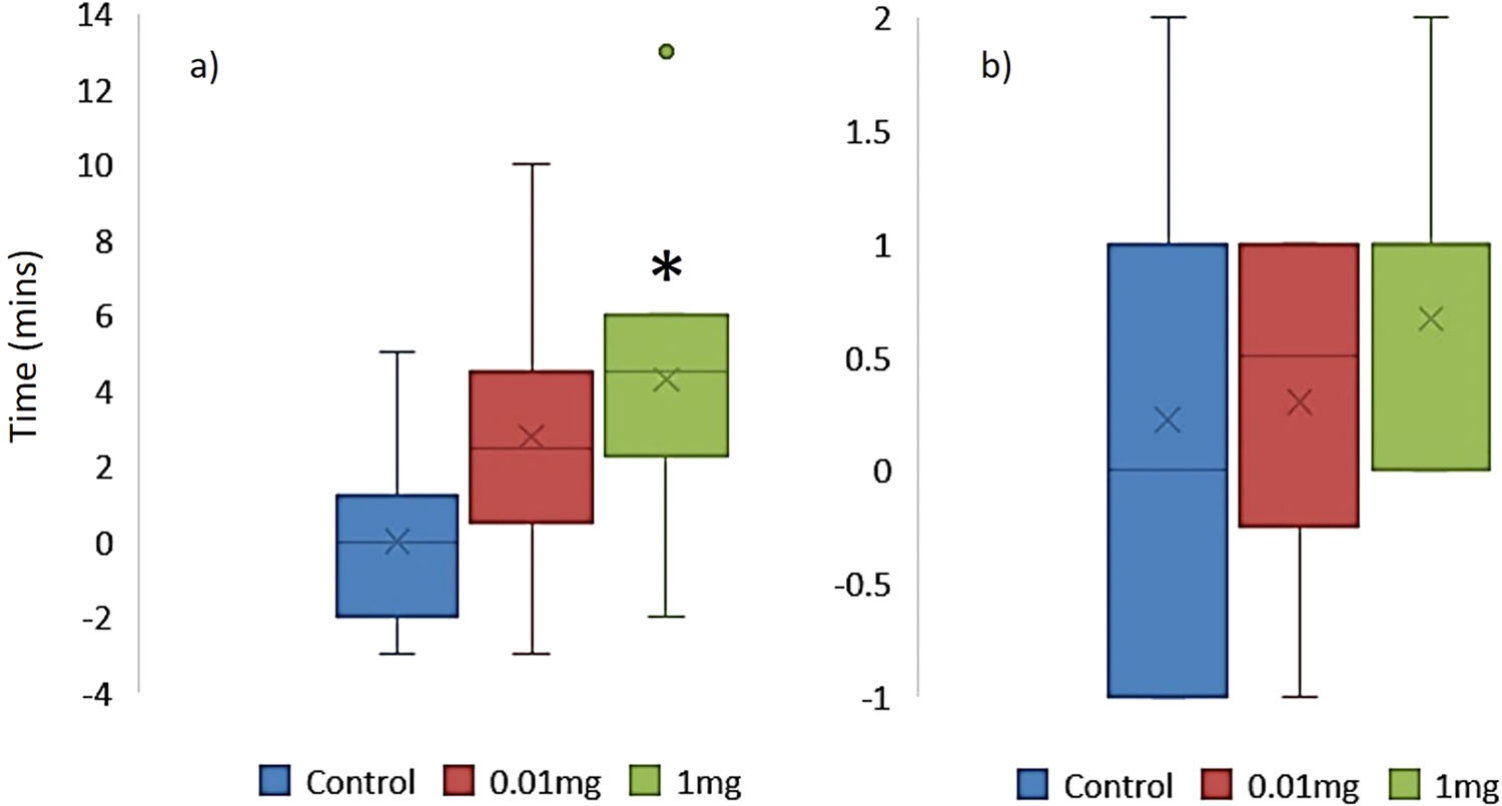
Mean difference in pre and post-exposure righting times (mins) of **a** *G. umbilicalis* and **b** *L. stagnalis* exposed over a 4 h period to 0, 0.01 mg L^−1^, and 1 mg L^−1^ fluoxetine hydrochloride (*x* = mean; line = median; bars = min/max; boxes = 25th/75th percentile). *Significantly different from control *p* < 0.05

Returning to the higher fluoxetine concentrations used in experiment 1, significant differences were observed in the proportion of *G. umbilicalis* snails, which detached from the tanks (Binary logistic regression: Wand = 7.594, df = 1, *p* = 0.006; Fig. 2). No snails detached from the tanks in the control and 0.01 mg L^−1^ fluoxetine exposure whilst 7 from 10 detached in the very high 1 mg L^−1^ exposures. Similarly, significant differences were also observed in the proportion of *L. stagnalis* snails, which detached from the tanks (Binary logistic regression: Wand = 4.523, df = 1, *p* = 0.032), whereby no snails detached in the control, 10% in the low concentration and 80% in the high concentration (Fig. 2). For those snails that did detach, no significant differences in mean detachment times were observed between snail species (Mann–Whitney, *p* > 0.05) with median times to detach being just below 2 h (Fig. 3). Pre and post-exposure righting times significantly and strongly correlated (Pearsons) with each other for both *G. umbilicalis* (*r* = 0.741; *p* < 0.001; Fig. 4a) and *L. stagnalis* (*r* = 0.861; *p* < 0.001; Fig. 4b). As a result the difference between the pre and post righting times were calculated and used to determine the effects of Flx exposure on righting times for the two snail species. Significant difference in righting time in *G. umbilicalis* were observed (Fig. 5; Kruskal–Wallis; *χ* = 7.636, df = 2, *p* = 0.022) but not for *L. stagnalis* (*p* > 0.05). Pairwise comparisons for *G. umbilicalis* found significant difference only between the control and high concentration (*p* = 0.02 adjusted for multiple comparisons). Significant differences were observed in the righting times between the two species (Mann–Whitney *U*, *p* < 0.001) pre and post-exposure with the marine species taking approximately three times longer (~10–12 min) compared to the freshwater species (~3 min).

**Fig. 6:**
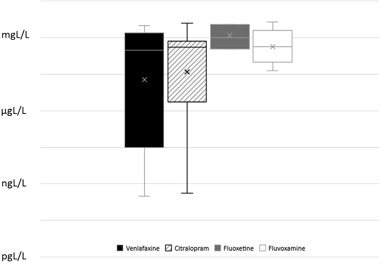
Box blot of variability in foot detachment in nine species of gastropod molluscs from published data using LOECs (Data from Fong and Hoy 2012; Fong and Molnar 2013 and this study; venlafaxine *n* = 7, citralopram *n* = 6, fluoxetine *n* = 7, fluvoxamine *n* = 5)

Meta-analysis of foot detachment LOEC values revealed no significant differences between antidepressants (Kruskal–Wallis 3.686, df = 3, *p* = 0.297) although median values were lower for venlafaxine and citralopram (Fig. 6). Overall median LOEC values for across all antidepressants were within the high µg L^−1^ to low mg L^−1^ range for the limited species done to date.

## Discussion

This study aimed to determine whether the SSRI fluoxetine could cause foot detachment and impact righting behaviour in two very distinct snail species representing the marine and freshwater environments. Previous studies have highlighted considerable variability between antidepressants and snail species. In this study, we only found significant effects on foot detachment and righting times at the high (1 mg L^−1^) concentration, which is much higher than might be expected from wastewater treatment plants (WWTP; Gardner et al. 2012). These results fall between those found by Fong and Molnar 2013 for fluoxetine who recorded LOECs of 345 μg L^−1^ for foot detachment for the marine snails, *Chlorostoma funebralis* and *Tegula fasciatus*, and 3.45 mg L^−1^ for *Nucella ostrina*, *Urosalpinx cinereal* and *Lithopoma americanum*.

The data collated on the nine species experimented for foot detachment for fluoxetine and fluvoxamine have median LOECs in the high µg L^−1^ to low mg L^−1^, which is within range of lethal concentrations (LC50s) for many invertebrate species (Brooks et al. 2003; Henry and Black 2007; Henry et al. 2004; Nałęcz-Jawecki et al. (2008)). We found no significant differences in the proportions of snails, which detached between the two marine and freshwater species or the time the two species took to detach. That is not to say that considerable differences could well occur in the ecophysiology of marine and freshwater snails and differences in the water chemistry can have profound effects on the potency of antidepressants (Nakamura et al. 2008; Valenti et al. 2009; Sundaram et al. 2015). Median LOECs venlafaxine and citralopram are also within the high µg L^−1^ range with some data reporting effects as low as ng–pg L^−1^ (Fong and Hoy 2012) suggesting great interspecies variability. Venlafaxine is an SNRI therefore might have different affinities for serotonin and norepinephrine transporters or indeed many other neurological receptors (Ford and Fong 2016). Citralopram on the other hand is a SSRI and arguably may have similar mode of action to the fluoxetine (i.e., inhibiting reuptake transporter proteins) although studies suggest variable receptor affinities between SSRIs (Stahl 1998). Whether this variability in studies is due to different species, drugs or experimental differences again is an interesting avenue for further investigation. Calow (1996) highlighted that there can be considerable differences between and within species. In our study the effects of fluoxetine on *G. umbilicalis* showed consistent results between repeats, in that only the higher concentration (1 mg L^−1^) resulted in any foot detachment albeit within subtle differences within experimental protocols.

For both species used in our study the snails which did detach did so in a median time of just under 2 h, which is similar (1–3 h) to the times taken by five marine species recorded by Fong and Molnar 2013. In our study the marine (*G. umbilicalis*) species did take approximately three times longer (10–12 min) to right itself compared to the freshwater species (~3 min; *L. stagnalis*). Whether this is simply down to the increased size of the *G. umbilicalis* vs. the *L. stagnalis*, physiological capacity to ‘right itself’ or a sensitivity difference between to the two species is unclear. Fong et al. (2017) recorded righting times of between 1–3 min for freshwater snail (*L. obsoleta*) in their study using a variety of different antidepressants.

Currently, very few long-term exposure studies exist in the effects of antidepressants on gastropod snails. However, within the Mollusca as a whole there are an increasing number of studies recording effects at environmentally relevant concentrations (Peters and Granek 2016; Bidel et al. 2016; Nentwig 2007; Gust et al. 2009; Franzellitti et al. 2014) and efforts have been made to devise Adverse Outcome Pathways related to predation and altered reproduction success (Fay et al. 2017). Our study, and those similar were conducted over a 4 h exposure period therefore one can only speculate what the long-term effects of these exposures might be on the behavioural or physiological status of these snails. Nentwig (2007) examined the effects of fluoxetine on reproductive endpoints in the New Zealand mud snail (*Potamopyrgus antipodarum*) over a longer exposure period (~2 months) and calculated an EC_10_ of 0.81 µg L^−1^ (based on measured concentrations) for reproduction endpoints. Interestingly, they report that during their experiments that snails in the high concentration (400 µg L^−1^) were immobile on the bottom of the test Beaker from the start of the experiment. They also mentioned that only a small percentage of the snails recovered and began grazing on food after a few days. Furthermore, nearly 90% (70/80 per replicate) remained immobile, and at day 56, and 100% mortality occurred in all replicates. The observation by Nentwig (2007) of snails lying motionless at the bottom of the tanks from the start of the experiments in the high concentrations was possibly ‘foot detachment’ as recorded by others (Fong and Hoy 2012; Fong and Molnar 2013; this study). This is interesting for a number of reasons, firstly, those individuals which did detach had very high levels of mortality over the approximate 2-month exposure period and secondly, their study predicts ‘effects’ at concentrations, which might be observed in sewage effluent. Sanchez-Argüello et al. (2009) in another long-term exposure recorded a stimulation of reproduction in freshwater snail (*Physa acuta*) at relatively low concentrations (nominal 31.25 and 62.5 µg L^−1^; recorded at 12 and 27 µg L^−1^) but a suppression of reproduction at higher concentrations (nominal 250 µg L^−1^; recorded 108 µg L^−1^) recorded over a 44-day exposure period. Gust et al. (2009) studied the effects of fluoxetine on reproduction in two species of freshwater snail (*Potamopyrgus antipodarum* and *Valvata piscinalis*). They emphasised the interspecies variability between the two species and also recorded a stimulation and non-monotonic concentration response curves in breeding for *Potamopyrgus antipodarum* with LOECs as low as 1 µg L^−1^. Non-monotonic concentration responses have been highlighted in a number of studies both within the invertebrates and vertebrates, with exposure to antidepressants (Guler and Ford 2010; Fong and Ford 2014; Ford and Fong 2016). However, they appear to be endpoint dependant with linear (monotonic) responses recorded for some biomarkers. All the studies on foot detachment and righting times have thus far appear monotonic in their response to antidepressants.

It is vital that results such as those presented here are published not based on their ‘impact’ in the sense that they are environmentally relevant but in order to determine the risk posed by environmental contaminants. This data we hope should aid those researchers and agencies in ascertaining the risk posed by these substances and the variability that appears to exist between species. Calow (1996) points out that whilst on one hand there is a need to understand, control and reduce variability in ecotoxicology, on the other hand there is a need to appreciate it and take it into account. This can only be done if researchers publish ‘all trials’ and not just the ones, which are significant or dramatic in their findings. There has been caution noted in the laboratory studies done on antidepressants to date due to various limitations in experimental designs and questions over repeatability (Sumpter et al. 2014). All of the current studies thus far detailing these behaviours (foot detachment and righting) have used nominal concentrations thus the actual concentrations maybe variable between experiments. Literature whereby actual vs. nominal concentrations has been measured have recorded the accuracy somewhere between ~30–100% (e.g., Nentwig 2007; Gust et al. 2009; De Castro-Català et al. 2017). Studies in our own labs have recorded fairly accurate nominal vs. actual concentrations from stocks, which quickly diminish to around 20–30% within 3 days (De Castro-Català et al. 2017). Nonetheless, the speed, relative low cost and repeatability of these experiments lends them well for high-throughput sublethal ecotoxicology studies if the downstream effects of such behaviours can be appropriately characterised.
